# Gender-Specific Association Between Perceived Stigma Toward Tuberculosis and Acceptance of Preventive Treatment Among College Students With Latent Tuberculosis Infection: Cross-Sectional Analysis

**DOI:** 10.2196/43972

**Published:** 2023-06-14

**Authors:** Yemin Yuan, Jin Jin, Xiuli Bi, Hong Geng, Shixue Li, Chengchao Zhou

**Affiliations:** 1 Centre for Health Management and Policy Research, School of Public Health Cheeloo College of Medicine Shandong University Jinan China; 2 Department of Epidemiology, School of Public Health Cheeloo College of Medicine Shandong University Jinan China; 3 Department of Prevention and Control Shandong Public Health Clinical Center Jinan China; 4 NHC Key Laboratory of Health Economics and Policy Research Shandong University Jinan China

**Keywords:** gender differences, perceived stigma, latent tuberculosis, treatment, acceptance, college students

## Abstract

**Background:**

With the increasing enrollment scale of colleges, the number of students on campus has risen sharply in China. The number of students with tuberculosis (TB) and rifampicin-resistant TB in colleges has increased significantly. Preventive treatment of latent tuberculosis infection (LTBI) is an important means for TB prevention and control in colleges. At present, the acceptance of LTBI treatment among college students remains unclear. In addition, evidence shows stigma may be one of the key factors affecting acceptance of LTBI treatment. To date, there is little direct evidence on the gender-specific association between perceived stigma toward TB and acceptance of LTBI treatment among college students.

**Objective:**

This study aimed to describe the acceptance of LTBI treatment among college students in an eastern province of China to explore the association between perceived stigma toward TB and acceptance of LTBI treatment and to examine the moderating effect of gender on the association.

**Methods:**

Data were derived from the project on the evaluation of LTBI treatment and its effectiveness among college students in Shandong, China. In total, 1547 college students were included in the analysis. We considered covariates at the individual and family levels. Multilevel mixed-effects logistic regression was used to examine the moderating role of gender and also explore the association between perceived stigma toward TB and acceptance of LTBI treatment.

**Results:**

The acceptance rate of LTBI treatment among the diagnosed college students was 46.7% (n=723). The proportion of female students (n=361, 51.5%) accepting LTBI treatment was higher than that of male students (n=362, 42.8%; *P*=.001). There was an interaction between perceived stigma toward TB and gender (OR 0.93, 95% CI 0.87-1.00; *P*=.06). Among college students with LTBI, perceived stigma toward TB was positively associated with acceptance of preventive treatment (OR 1.03, 95% CI 1.00-1.08, *P*=.05). Perceived stigma toward TB was positively associated with accepting LTBI treatment only among male students (OR 1.07, 95% CI 1.02-1.12; *P*=.005).

**Conclusions:**

The acceptance rate of preventive treatment among college students with LTBI was low. Contrary to our expectations, perceived stigma toward TB was positively associated with acceptance of preventive treatment. Gender moderated this association; high perceived stigma toward TB was associated with acceptance of preventive treatment only in male gender. Gender-specific strategies are effective in improving the acceptability of LTBI treatment in colleges.

## Introduction

According to the Global Tuberculosis Report 2022, China has the third highest number of cases among 30 countries with a high burden of tuberculosis (TB). In China, the reported incidence of TB among students dropped from 20.6/100,000 enrolled students in 2010 to 13.4/100,000 in 2015 but increased to 17.5/100,000 in 2019 [1]. In 2019, TB cases in students accounted for 6.2% of all TB cases [2]. In recent years, the annual incidence of pulmonary TB among college students is higher than the national level for the same age group [3]. A systematic review and meta-analysis of TB outbreaks among students in mainland China showed that the incidence of TB was higher in colleges compared to high schools [4]. The number of students with TB and rifampicin-resistant TB increased significantly in China from 2015 to 2019, most of whom were college students [5]. With the expansion of national college enrollment and more schools being open, the prevention and control of TB in colleges have become challenging.

Latent tuberculosis infection (LTBI) is defined as a state of persistent immune response to stimulation by *Mycobacterium tuberculosis* antigens with no evidence of clinically manifest active TB [6]. Approximately 350 million people in China have an infection caused by *Mycobacterium tuberculosis* [7], which means that a considerable number of people are in the LTBI state. The population with LTBI is a large reservoir of TB and has a 5%-10% lifetime chance of developing active TB if LTBI is untreated [8]. LTBI treatment is one of the important interventions to finally meet the end TB targets. TB outbreaks still occur from time to time in Chinese colleges. LTBI treatment is an important means for TB prevention and control in colleges. The acceptance of LTBI treatment among college students remains unclear at present. Clarification of the LTBI treatment acceptance and identification of the key factors influencing it may be of great significance to implement this intervention among college students with LTBI.

Stigma is defined as “a social process, experienced or anticipated, characterized by exclusion, rejection, blame or devaluation that results from experience, perception or reasonable anticipation of an adverse social judgement about a person or group” [9]. TB-related stigma, including fear of infection, may be a barrier to initiation and successful completion of LTBI treatment [10,11]. A qualitative study in the Netherlands [12] found that the difficulty to differentiate LTBI from TB and the consequent fear of TB infection and disease had negatively impacted the screening or treatment of LTBI among asylum seekers and refugees. Another study in England [13] indicated that migrants in a college environment were reluctant to accept LTBI screening and treatment programs because they were concerned about reputational loss and stigma of being involved in a TB project. These findings indicate that stigma may be one of the key factors affecting acceptance of LTBI treatment. Based on existing findings, we hypothesize that perceived stigma toward TB is negatively associated with acceptance of preventive treatment among college students with LTBI.

Globally, many more adult men than women have been diagnosed with TB and have died from it [14]. A meta-analysis reported that men were disadvantaged in seeking and accessing TB care and suggested that men were a high-risk group requiring improved access to TB care [15]. In addition, studies found that substantial gender differences in the epidemiologic burden of TB were almost solely attributable to gender differences in disease incidence and treatment initiation, in both of which men were disadvantaged [16]. Studies in Nigeria and Australia showed that there were gender differences in TB diagnosis, treatment, and outcomes [17,18]. Gender differences in TB may be greater in China. The average male to female proportion in China was about 2.19:1, which was higher than the global average level (1.9:1) [19,20]. However, there are few studies on gender differences in LTBI-related aspects. We hypothesize that gender differences have an impact on the association between perceived stigma toward TB and the acceptance of LTBI treatment.

Given the importance of LTBI treatment, TB control in schools, and the critical role of stigma, the aim of this study was (1) to describe the acceptance of LTBI treatment among college students in an eastern province of China; (2) to explore the association between perceived stigma toward TB and the acceptance of LTBI treatment; and (3) to examine the moderating effect of gender on this association.

## Methods

### Study Setting and Participants

This study used data from the project on treatment and effectiveness evaluation of LTBI among college students in Shandong province, China. More information about procedures has been described in detail in our previous publication [21]. The study began in September 2020 and was conducted in 6 cities (Jinan, Qingdao, Yantai, Rizhao, Linyi, and Zibo) using a cluster random sampling method to select samples. We selected students aged ≥18 years with positive purified protein derivative test results (ie, induration diameter ≥10 mm or accompanied by blisters, papules, double circles, and lymphatitis) to participate in our questionnaire survey. We excluded patients previously diagnosed with active pulmonary TB or extrapulmonary TB as well as those with a history of mental illness or epilepsy. Respondents were instructed to fill out the electronic survey questionnaire by trained graduate students to ensure the quality of the questionnaire. In total, 1691 individuals were eligible, and 1631 completed the whole survey.

We used the following formula to calculate the sample size:



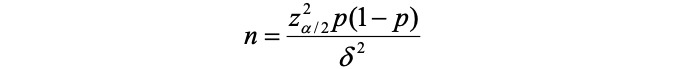



In this formula, *n, δ,* and *p* are estimations for sample size, allowable error, and population rate *π*, respectively. Because acceptance of LTBI treatment among college students with LTBI is not clear in China, we used the positive prevalence of the tuberculin skin test (TST) to calculate the sample size. The prevalence of positive TSTs in Chinese college students using 10mm cutoff value was 9%-49% [22,23]. We set *P*=.09, *α*=.05 (2-tailed), and *δ*=0.009. Considering the sampling method and the 20% lost follow-up rate, the TST screening sample was finally determined to include 20,330 people [21].

### Ethical Considerations

This study protocol received ethics approval from the Ethical Committee of the School of Public Health in Shandong University (LL20200306). The investigation was conducted after the acquisition of written informed consent from all participants.

### Measures

#### Acceptance of LTBI Treatment

Acceptance of LTBI treatment was measured by the question “Will you accept preventive treatment of latent tuberculosis infection?” The answer was divided into either accepting treatment or refusing treatment. We eliminated 84 samples whose answers were inconclusive. Finally, 1547 college students were included in the analysis.

#### Perceived Stigma Toward TB

Perceived stigma toward TB was assessed using 11 questions [24]. These problems originate in countries with a high burden of TB. More details about these questions are described in Multimedia Appendix 1. The answer to each question included “Yes” and “No.” “Yes” indicated the presence of perceived stigma, and “No” indicated the absence of perceived stigma. The score ranged from 0 to 11. The higher the score, the higher the perceived stigma toward TB. The Cronbach coefficient was .880 in this study.

#### Covariates

In this study, gender was used as a moderating variable. Based on previous research [25,26], some other factors may also influence the acceptance of LTBI treatment. Therefore, we added covariates such as age (continuous), type of student (eg, medical students and nonmedical students), type of residence (eg, urban and rural), parents’ levels of education (eg, primary school or below, junior high school, and high school or above), household income in 2019 (eg, quintile 1, quintile 2, quintile 3, and quintile 4), smoking (yes), current drinking (yes), physical activity level (eg, low, moderate, and high), exposure history of TB (eg, no, yes, or unknown), boarding experiences (yes), and knowledge of TB (continuous). Physical activity level was measured by the Chinese version of International Physical Activity Questionnaire Short Form [27]. The score of TB knowledge ranges from 0 to 45 (Multimedia Appendix 2). The higher the score, the higher the level of TB knowledge.

### Statistical Analysis

First, a descriptive analysis was used to describe participants’ characteristics with mean (SD) or median (IQR) values for continuous variables. Frequency and percentage values were used to describe categorical variables. Second, Pearson chi-square test (for categorical variables) and 2-tailed student *t* test or Wilcoxon rank-sum test (for continuous variables) were used to compare characteristics of male and female students. Then, since 1547 participants came from 6 cities and 16 colleges, multilevel mixed-effects logistic regression (Multimedia Appendix 3) was performed to examine the moderating role of gender. We added the multiplicative interaction term (perceived stigma toward TB × gender) in models. The association between perceived stigma toward TB and acceptance of LTBI treatment was also assessed by multilevel mixed-effects logistic regression. In model 1, no covariate was included. Model 2 was based on model 1, with additional adjusting for age, type of student, type of residence, father’s level of education, mother’s level of education, household income, smoking, current drinking, physical activity level, exposure history of TB, boarding experiences, and knowledge of TB. Odds ratios (ORs) and 95% CIs were presented as measures of effect. A 2-tailed *P*<.1 was considered as statistically significant. We conducted all analyses in Stata (version 14.2; Stata Corp).

## Results

Table 1 shows the baseline characteristics of participants by gender. Of the 1547 participants, about 846 (54.7%) were male students. The average age of respondents was 18.5 (SD 0.8) years, and most of them were nonmedical students. The proportion of female medical specialty students was higher than that of male students (*P*<.001). In terms of lifestyle behaviors, male students had higher proportions of smoking and current drinking than female students (*P*<.001). The average TB knowledge score was 19 (SD 7.7). The level of TB knowledge in female students was higher than that in male students (*P*=.05). The median score of perceived stigma toward TB was 1 (IQR 0-4), with a statistical difference between male and female students (*P*=.09). A total of 723 (46.7%) students with LTBI were willing to accept preventive treatment. The proportion of female students accepting LTBI treatment was higher than that of male students (*P*=.001). Male students who accepted LTBI treatment had higher perceived stigma score compared to those who did not (*P*=.02; Multimedia Appendix 4).

The interaction between perceived stigma toward TB and gender and its effect on acceptance of LTBI treatment is shown in Table 2. After adjusting for covariates, there was an interaction between perceived stigma toward TB and gender (OR 0.93, 95% CI 0.87-1.00; *P*=.06).

**Table 1:** Participants’ characteristics of latent tuberculosis infection (LTBI) college students by gender in Shandong, China, in 2020.

Variables		Total, n (%)	Male, n (%)	Female, n (%)	*P* value
Total		1547 (100)	846 (54.7)	701 (45.3)	
Age (years), mean (SD)		18.5 (0.8)	18.6 (0.8)	18.5 (0.8)	.25
**Type of students, n (%)**					<.001
	Medical students	247 (16)	82 (9.7)	165 (23.5)	
	Nonmedical students	1300 (84)	764 (90.3)	562 (76.5)	
**Type of residence, n (%)**					.28
	Urban	740 (47.8)	394 (46.6)	346 (49.4)	
	Rural	807 (52.2)	452 (53.4)	355 (50.6)	
**Father’s level of education, n (%)**					.75
	Primary school or below	268 (17.3)	141 (16.7)	127 (18.1)	
	Junior high school	721 (46.6)	397 (46.9)	324 (46.2)	
	High school or above	558 (36.1)	308 (36.4)	250 (35.7)	
**Mother’s level of education, n (%)**					.24
	Primary school or below	467 (30.2)	248 (29.3)	219 (31.2)	
	Junior high school	650 (42)	348 (41.1)	302 (43.1)	
	High school or above	430 (27.8)	250 (29.6)	180 (25.7)	
**Household income^a^, n (%)**					.74
	Quartile 1	426 (27.5)	231 (27.3)	195 (27.8)	
	Quartile 2	394 (25.5)	211 (24.9)	183 (26.1)	
	Quartile 3	376 (24.3)	203 (24)	173 (24.7)	
	Quartile 4	351 (22.7)	201 (23.8)	150 (21.4)	
Smoking (yes)		159 (10.3)	151 (17.9)	8 (1.1)	<.001
Current drinking (yes)		382 (24.7)	324 (38.3)	58 (8.3)	<.001
**Physical activity level, n (%)**					.26
	Low	558 (36.1)	306 (36.2)	252 (36)	
	Moderate	509 (32.9)	265 (31.3)	244 (34.8)	
	High	480 (31)	275 (32.5)	205 (29.2)	
**Exposure history of TB^b^, n (%)**					.68
	No	1,093 (70.6)	596 (70.5)	497 (70.9)	
	Yes	58 (3.8)	35 (4.1)	23 (3.3)	
	Unknown	396 (25.6)	215 (25.4)	181 (25.8)	
Boarding experiences (yes), n (%)		1264 (81.7)	700 (82.7)	564 (80.5)	.25
Knowledge of TB (score), mean (SD)		19.0 (7.7)	18.7 (7.9)	19.5 (7.4)	.05
Perceived stigma toward TB (score), median (IQR)		1 (4)	1 (4)	1 (4)	.09
Acceptance of LTBI treatment (yes), n (%)		723 (46.7)	362 (42.8)	361 (51.5)	.001

^a^Quartile 1: ≤US $5800; quartile 2: >US $5800 and ≤US $10,150; quartile 3: >US $ 10,150 and ≤14,500; quartile 4: >US $ 14,500. Average exchange rate in 2019: US $ 1=RMB 6.8967.

^b^TB: tuberculosis.

**Table 2:** The effect of gender on the cross-sectional interaction between perceived stigma toward tuberculosis (TB) and acceptance of latent tuberculosis infection (LTBI) treatment among college students in Shandong, China, in 2020.

Variables	Model 1^a^				Model 2^b^			
	OR^c^	SE	95% CI	*P* value	OR	SE	95% CI	*P* value
Perceived stigma toward TB	1.06	0.025	1.01-1.11	.005	1.07	0.025	1.02-1.12	.005
Gender (female)	1.80	0.261	1.36-2.39	<.001	1.94	0.300	1.43-2.62	<.001
Perceived stigma toward TB × gender	0.94	0.035	0.87-1.00	.07	0.93	0.035	0.87-1.00	.06

^a^Model 1 was unadjusted.

^b^Model 2 was adjusted for age, type of student, type of residence, father’s level of education, mother’s level of education, household income, smoking, current drinking, physical activity level, TB exposure history, boarding experiences, and knowledge of TB.

^c^OR: odds ratio.

Table 3 presents the relationship between perceived stigma toward TB and acceptance of LTBI treatment among male and female students. Perceived stigma toward TB was positively associated with acceptance of preventive treatment among college students with LTBI (OR 1.03, 95% CI 1.00-1.08; *P*=.05). Among male students, high perceived stigma toward TB was associated with accepting LTBI treatment when compared to low perceived stigma toward TB (OR 1.07, 95% CI 1.02-1.12; *P*=.005). However, there was no association between perceived stigma toward TB and acceptance of LTBI treatment among female students.

**Table 3:** Cross-sectional association between perceived stigma toward tuberculosis (TB) and acceptance of latent tuberculosis infection (LTBI) treatment among male and female students in Shandong, China, in 2020.

Variables	Model 1^a^				Model 2^b^			
	OR^c^	SE	95% CI	*P* value	OR	SE	95% CI	*P* value
Perceived stigma toward TB (n=1547)	1.04	0.019	1.00-1.07	.04	1.03	0.019	1.00-1.08	.05
Perceived stigma toward TB in male students (n=846)	1.06	0.025	1.02-1.11	.005	1.07	0.026	1.02-1.12	.005
Perceived stigma toward TB in female students (n=701)	1.00	0.029	0.94-1.06	.93	0.99	0.030	0.94-1.05	.87

^a^Model 1 was unadjusted.

^b^Model 2 was adjusted for age, type of students, type of residence, father’s level of education, mother’s level of education, household income, smoking, current drinking, physical activity level, exposure history of TB, boarding experiences, and knowledge of TB.

^c^OR: odds ratio.

## Discussion

### Principal Findings

In this study, only 723 (46.7%) college students diagnosed with LTBI were willing to accept preventive treatment. This figure is lower than what has been previously reported for other students’ acceptance rates of LTBI treatment, ranging between 63.9% and 87% in China [28,29]. The possible reason is that participants in this study were general college students, while previous research focused on students who had close contacts with TB and was related to the screening and management of students’ close contacts with TB [30]. For close contacts, schools actively organized screening and recommend LTBI treatments. Close contacts were more aware of TB than the student and more likely to accept LTBI treatment for fear of developing TB.

Our study observed a higher acceptance rate of LTBI treatment among female students than male students, contrary to previous studies in the United States and Canada [31]. One possible explanation for our findings is that female students have a higher level of TB knowledge than male students. Previous studies found that patients with LTBI who had low TB knowledge scores in student TB contacts or TB-designated hospitals were less likely to accept preventive treatment [28,32]. Students with higher knowledge of TB had some knowledge of the spread, harm, and prevention of TB. They wanted to reduce their risk of developing TB. When health workers recommended LTBI treatment, female students were more likely to accept it.

Our results showed that higher perceived stigma toward TB was positively related to acceptance of preventive treatment among college students with LTBI. This did run counter to our initial hypothesis, wherein we assumed that perceived stigma toward TB may be negatively associated with acceptance of LTBI treatment. No direct evidence has shown this association among college students, but prior studies found that TB-related stigma was negatively associated with LTBI treatment acceptance in other populations (eg, refugees and migrants) [12,13]. On the one hand, we speculate that the difference is due to people’s surroundings. College students live in a trustful and inclusive environment. College students did not score high on perceived stigma toward TB in our study, suggesting that they did not have embedded fears surrounding TB. Of course, it could also be that they did not know much about TB. After all, the TB knowledge score of college students in the study was 19.0 out of 45. However, TB stigma is relatively high in the community. Immigrants may feel singled out and stigmatized for other reasons [33,34]. One study in Eritrea [35] showed that restrained contacts due to fear of getting infected with TB, gossiping, and finger pointing in the community caused enacted and anticipated stigma among patients with TB. These findings indicate that the relationship between perceived stigma toward TB and acceptance of LTBI treatment may be quite opposite in different populations. On the other hand, LTBI is still different from TB infection. Although participants experienced enforced stigma, which could impede treatment initiation, their strong motivation to prevent becoming sick with TB resulted in acceptance of LTBI treatment.

It is worth noting that perceived stigma toward TB was positively associated with acceptance of LTBI treatment in male students, but there was no association between the stigma toward Tb and LTBI treatment acceptance in female students. Similar to previous studies on the impact of gender differences on TB epidemiological characteristics and access to services [15,16], we found gender differences played a part in the association between perceived stigma toward TB and acceptance of LTBI treatment. One possible factor that accounts for the disparity could be the different temperament and personality characteristics of male and female students. Women tend to be cautious, careful, worried, or pessimistic; men are more carefree, relaxed, and upbeat, even when most people are worried [36,37]. Therefore, male students may be more likely to accept LTBI treatment after feeling perceived stigma toward TB, whereas female students are not. Another plausible explanation is that the differences are related to men’s unhealthy behaviors, such as smoking and drinking. Studies found men were overrepresented in various pulmonary TB risk groups, notably alcoholic and tobacco smokers [16,38]. In this study, the proportion of male students who smoke and drink alcohol far exceeded that of female students. Despite higher perceived TB stigma, male students with LTBI were more likely to accept preventive treatment for fear of developing active TB. However, these pathways are speculative. Future research can explore the underlying mechanisms of the relationship between perceived stigma toward TB and LTBI treatment acceptance.

Perceived stigma toward TB was not associated with acceptance of LTBI treatment in female students. The reason may be that perceived stigma is not a key factor in female students accepting preventive treatment. In our study, the proportion of female medical specialty students was higher than that of male students. Moreover, female students have similar stigma scores but higher TB knowledge and LTBI treatment acceptance scores compared to male students. Of the added covariates, we found that exposure history of TB (Yes: OR 3.00, 95% CI 0.98-9.16; *P*=.05 and Unknown: OR 1.61, 95% CI 1.10-2.37; *P*=.02) and TB knowledge (OR 1.02, 95% CI 1.00-1.05; *P*=.08) were positively associated with acceptance of LTBI treatment among female students. In the future, research may focus on the factors influencing the acceptance of preventive treatment in women.

The current findings have some policy implications. Gender may be an important heterogeneous factor influencing the acceptance of LTBI treatment in colleges. It is recommended that colleges regularly and continuously carry out various forms of TB and LTBI knowledge publicity and education activities to raise students’ awareness of TB. The perceived stigma toward TB among male students needs to be addressed in improving their treatment acceptance. It is suggested that colleges provide suitable psychological counseling for male students. Overall, perhaps gender-specific management can improve the acceptability of LTBI treatment among college students.

This study has some limitations. First, the results were based on colleges in Shandong Province. Our findings were not necessarily generalizable to other settings within or outside China. Moreover, cross-sectional data used in this study were impossible to demonstrate the causality between perceived stigma toward TB and acceptance of LTBI treatment. Establishing and analyzing a multiyear longitudinal cohort of colleges covering different geographic regions in future studies could better elucidate the impact of gender on the relationship between perceived stigma toward TB and willingness for LTBI treatment.

### Conclusions

In conclusion, we found that the acceptance rate of preventive treatment among college students with LTBI was low. The acceptance rate of female students was higher than that of male students. There were gender differences in the association between perceived stigma toward TB and acceptance of LTBI treatment. Only among male students, those with high perceived stigma toward TB were more likely to accept LTBI treatment. This study implies that gender-specific strategies in colleges may be effective in improving the acceptability of preventive treatment.

## Appendix

Multimedia Appendix 1 Perceived stigma toward tuberculosis.

Multimedia Appendix 2 Knowledge of tuberculosis.

Multimedia Appendix 3 An introduction of multilevel mixed-effects logistic regression.

Multimedia Appendix 4 The prevalence of acceptance of LTBI treatment in Shandong, China, in 2020.

## Abbreviations

LTBI: latent tuberculosis infection
OR: odds ratio
TB: tuberculosis
TST: tuberculin skin test

## Data Availability

The data sets used and analyzed during this study can be obtained from the corresponding author upon request.
