# Supplementary material for: Gender-Specific Association Between Perceived Stigma Toward Tuberculosis and Acceptance of Preventive Treatment Among College Students With Latent Tuberculosis Infection: Cross-Sectional Analysis
Source: JMIR Public Health Surveill. 2023 Jun 14;9:e43972. doi: 10.2196/43972 (PMC10337361; doi:10.2196/43972)
Supplement: Multimedia Appendix 1 [file publichealth_v9i1e43972_app1.doc]

**Supplementary file 1: Perceived stigma towards tuberculosis**

1. If you had TB, others would think less of you.

1=Yes 2=No

2. if you find out you had TB, you would feel ashamed and embarrassed.

1=Yes 2=No

3. If you find out you have TB, you would think less of you.

1=Yes 2=No

4. If you had TB, others would avoid you.

1=Yes 2=No

5. If you had TB, you would have a problem of finding a partner even after cure.

1=Yes 2=No

6. If you had TB, your partner would refuse to have sex with you.

1=Yes 2=No

7. If you had TB, you would be asked to stay away from a social group.

1=Yes 2=No

8. If you had TB, you would not disclose your status to anyone.

1=Yes 2=No

9. If you had TB, you would affect others by the disease.

1=Yes 2=No

10. If you had TB, others would think less of your family.

1=Yes 2=No

11. If you had TB, it would be a problem for your children.

1=Yes 2=No
