# Supplementary material for: Gender-Specific Association Between Perceived Stigma Toward Tuberculosis and Acceptance of Preventive Treatment Among College Students With Latent Tuberculosis Infection: Cross-Sectional Analysis
Source: JMIR Public Health Surveill. 2023 Jun 14;9:e43972. doi: 10.2196/43972 (PMC10337361; doi:10.2196/43972)
Supplement: Multimedia Appendix 3 [file publichealth_v9i1e43972_app3.doc]

**Supplementary file 3: An introduction of multilevel mixed-effects** **logistic regression**

Mixed effects logistic regression is used to model binary outcome variables, in which the log odds of the outcomes are modeled as a linear combination of the predictor variables when data are clustered or there are both fixed and random effects. Multilevel mixed-effects logistic regression deals with data with hierarchical structure characteristics (eg. village - household - individual). Individuals have a certain degree of similarity or aggregation in terms of economic level, lifestyle and living habits. Individual data are not independent. Multilevel mixed-effects logistic regression can decompose the random error term in the traditional logical regression to the level corresponding to the data hierarchy, making the individual random error more pure.

Multilevel mixed-effects logistic regression allows for many levels of random effects [1]. However, for simplicity, for now we consider the two-level model, where for a series of M independent clusters, and conditional on a set of random effects **u***_j_*,

$\text{Pr}(y_{ij}=1|\text{x}_{ij},\text{u}_{j})=H(\text{x}_{ij}\beta+\text{z}_{ij}\text{u}_{j})$ (1)

for *j* = 1, . . . , *M* clusters, with cluster *j* consisting of *i* = 1, . . . , *n_j_* observations. The responses are the binary-valued *y_ij_* , and we follow the standard Stata convention of treating *y_ij_* = 1 if *depvar_ij_* ≠ 0 and treating *y_ij_* = 0 otherwise. The 1 × *p* row vector x*_ij_* are the covariates for the fixed effects, analogous to the covariates you would find in a standard logistic regression model, with regression coefficients (fixed effects) *β*. For notational convenience here and throughout this manual entry, we suppress the dependence of *y_ij_* on x*_ij_*.

The 1 × *q* vector z*_ij_* are the covariates corresponding to the random effects and can be used to represent both random intercepts and random coefficients. For example, in a random-intercept model, z*_ij_* is simply the scalar 1. The random effects **u***_j_* are *M* realizations from a multivariate normal distribution with mean **0** and *q* × *q* variance matrix **Σ**. The random effects are not directly estimated as model parameters but are instead summarized according to the unique elements of **Σ**, known as variance components. One special case of (1) places

z**_ij_** = x*_ij_* so that all covariate effects are essentially random and distributed as multivariate normal with mean *β* and variance **Σ**.

Finally, because this is logistic regression, *H*(·) is the logistic cumulative distribution function, which maps the linear predictor to the probability of a success (*y_ij_* = 1), with $H(v)=\exp(v)/\{1+\exp(v)\}$.

Model (1) may also be stated in terms of a latent linear response, where only *y_ij_* = *I*($y_{ij}^{*}$> 0) is observed for the latent

$$y_{ij}^{*}=\text{x}_{ij}+\text{z}_{ij}\text{u}_{j}+\varepsilon_{ij}$$

The errors $\varepsilon_{ij}$ are distributed as logistic with mean 0 and variance π^2^/3 and are independent of **u***_j_* .

Example: Three-level random-intercept model

Rabe-Hesketh, Toulopoulou, and Murray analyzed data from a study measuring the cognitive ability of patients with schizophrenia compared with their relatives and control subjects [2]. Cognitive ability was measured as the successful completion of the “Tower of London”, a computerized task, measured at three levels of difficulty. For all but one of the 226 subjects, there were three measurements (one for each difficulty level). Because patients’ relatives were also tested, a family identifier, family, was also recorded.

We fit a logistic model with response dtlm, the indicator of cognitive function, and with covariates difficulty and a set of indicator variables for group, with the controls (group==1) being the base category. We allow for random effects due to families and due to subjects within families, and we request to see odds ratios.

“melogit dtlm difficulty i.group || family: || subject: , or”.

This is a three-level model with two random-effects equations, separated by ||. The first is a random intercept (constant only) at the family level, and the second is a random intercept at the subject level. The order in which these are specified (from left to right) is significant—melogit assumes that subject is nested within family. The above extends to models with more than two levels of nesting by adding more random-effects equations, each separated by ||. The order of nesting goes from left to right as the groups go from biggest (highest level) to smallest (lowest level).

**References**

1. [Stata multilevel mixed-effects reference](https://www.stata.com/manuals13/me.pdf) manual release 13. College Station, Texas. A Stata Press Publication. 2013. <https://www.stata.com/manuals13/me.pdf>

2. Rabe-Hesketh S, Toulopoulou T, Murray RM. Multilevel modeling of cognitive function in schizophrenic patients and their first degree relatives. Multivariate Behav Res 2001 Apr;36(2):279-298.
