# Supplementary material for: Gender-Specific Association Between Perceived Stigma Toward Tuberculosis and Acceptance of Preventive Treatment Among College Students With Latent Tuberculosis Infection: Cross-Sectional Analysis
Source: JMIR Public Health Surveill. 2023 Jun 14;9:e43972. doi: 10.2196/43972 (PMC10337361; doi:10.2196/43972)
Supplement: Multimedia Appendix 4 [file publichealth_v9i1e43972_app4.doc]

**Supplementary file 4: Table S1.** The prevalence of acceptance of LTBI treatment in Shandong, China, 2020.

| Variables | No acceptance | Acceptance | *P*-value |
| --- | --- | --- | --- |
| All (n=1,547), n (%) | 824(53.3) | 723(46.7) |  |
| Perceived stigma towards TB (score), median (IQR) | 1(4) | 1(4) | .066 |
| Male students (n=846), n (%) | 484(57.2) | 362(42.8) |  |
| Perceived stigma towards TB (score), median (IQR) | 1(4) | 1(4) | .018 |
| Female students (n=701), n (%) | 340(48.5) | 361(51.5) |  |
| Perceived stigma towards TB (score), median (IQR) | 1(4) | 1(4) | .857 |

TB, tuberculosis; LTBI, latent tuberculosis infection; IQR, interquartile range.
